# Declining Diagnostic Accuracy of the Laboratory Risk Indicator for Necrotizing Fasciitis (LRINEC) Score in Necrotizing Fasciitis: A Case Report With Contemporary Evidence Review

**DOI:** 10.7759/cureus.102159

**Published:** 2026-01-23

**Authors:** Micah Pippin, Stephanie Nguyen, Sanjay Shrestha

**Affiliations:** 1 Family Medicine, Louisiana State University Health Sciences Center, Alexandria, USA; 2 Family Medicine, Rapides Regional Medical Center, Alexandria, USA

**Keywords:** lower limb wounds, lrinec score, necrotizing fasciitis (nf), necrotizing soft-tissue infection, soft-tissue infection

## Abstract

Necrotizing fasciitis is a rapidly advancing soft-tissue infection with high morbidity and mortality, and markedly worse prognosis when complicated by delayed diagnosis and intervention. Distinguishing necrotizing fasciitis from less severe soft-tissue infections remains a significant clinical challenge, prompting the development of the Laboratory Risk Indicator for Necrotizing Fasciitis (LRINEC) score to aid early recognition. Early enthusiasm for the score led to widespread clinical use; however, subsequent experience has raised concerns about its utility as a sensitive rule-out tool. We present a case of surgically confirmed necrotizing fasciitis in which the LRINEC score failed to appropriately categorize the condition as high risk at presentation, prompting a focused investigation of evolving LRINEC literature. This case and accompanying review highlight the limitations of the LRINEC score as an isolated rule-out diagnostic instrument and support its more adjunctive role to clinical judgement, physical examination, imaging, and early surgical consultation. A high index of suspicion combined with conscientious implementation of comprehensive and inclusive diagnostic utilities can avoid delays in diagnosis and promote improved outcomes while modified and novel diagnostic applications are investigated.

## Introduction

Necrotizing fasciitis is an invasive soft-tissue infection recognized for its fascial necrosis, systemic toxicity, high mortality, and the importance of prompt recognition and early surgical intervention. At presentation, clinical findings may be subtle and overlap with those of more benign soft tissue infections, creating a challenging diagnostic conundrum and necessitating the development and implementation of effective methods for early identification. In response to this dilemma, Wong et al. developed the Laboratory Risk Indicator for Necrotizing Fasciitis (LRINEC) score in 2004, utilizing routine laboratory parameters to discern the presence of necrotizing fasciitis from its more innocuous differentials [1]. Wong et al.’s derivation study and subsequent validation reviews seemed to substantiate the score’s diagnostic accuracy and solidified its role in decision-making algorithms and clinical teaching [1-5]. Despite early promise, well-designed contemporary investigations and systematic reviews have reported more modest results, specifically, demonstrating substantially lower sensitivity and prompting reconsideration of LRINEC’s value as a screening tool [6-10].

We present a case of necrotizing fasciitis notable for yielding a low-risk LRINEC score despite significant clinical disease. Using this case as an impetus, we track the progress of LRINEC’s performance over time, examine factors contributing to declining sensitivity, discuss appropriate utilization of LRINEC in the context of a comprehensive clinical, radiographic, and surgical evaluation, and briefly introduce alternative diagnostic tools currently being investigated.

## Case presentation

A 49-year-old female presented to the emergency department with complaints of worsening bilateral leg swelling and pain associated with deterioration of her chronic lower extremity wounds and ulcers. The pain had been persistent and progressively intensifying over the previous several days. She described the pain as constant and severe, rating her discomfort as 8 on a pain scale of 1 to 10. The pain was exacerbated by standing and ambulation and partially relieved by rest, but no patient-directed pharmacologic analgesia had been attempted. She reported associated red to purple discoloration of the lower extremities, especially the feet, with fluid drainage from her chronic wounds and peeling skin, but no localized temperature change. No extremity weakness or paresthesia was endorsed. The patient confirmed generalized fatigue and subjective fevers, but no chills, rigors, or night sweats. Symptom reporting from other systems was mostly negative, with no cardiac, respiratory, neurologic, or gastrointestinal elements described by the patient.

Past medical history included chronic bilateral lower extremity ulcers for the past two to three years. The etiology of her ulcers had never been distinguished from venous insufficiency, peripheral artery disease, neuropathy, or some other syndrome. Her history was otherwise ambiguous, likely due to infrequent engagement with the medical system. She was unemployed with unstable housing and a history of methamphetamine use disorder. She smoked cigarettes daily; however, she could not accurately report the number of pack-years. No alcohol use was endorsed. She denied any surgical history, and her family history was unknown. No known drug allergies were identified.

**Figure 1:**
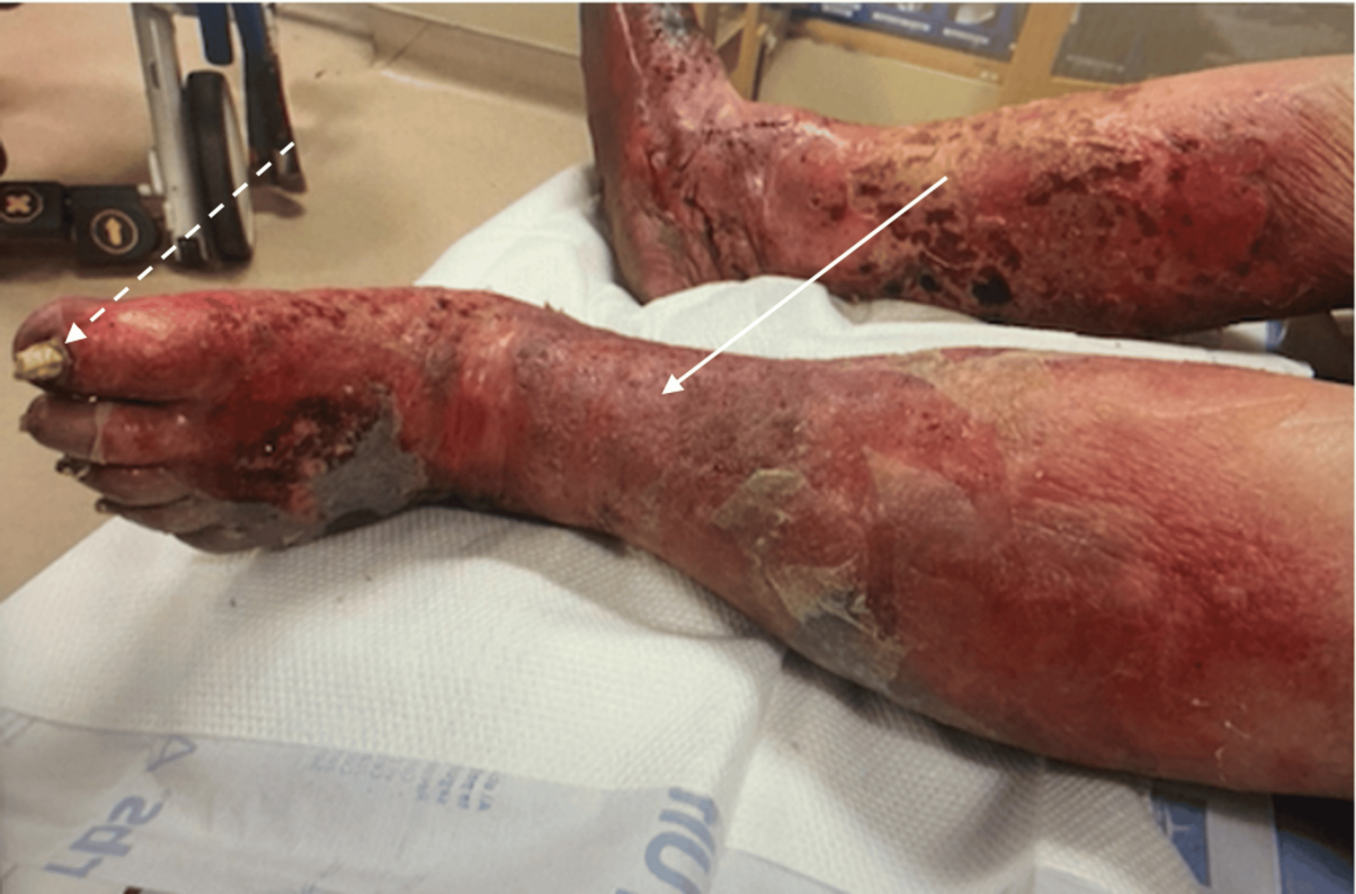
Diffuse erythema, dusky discoloration, tense edema, and lipodermatosclerosis (solid arrow) with hypertrophic nails (dashed arrow)

**Figure 2:**
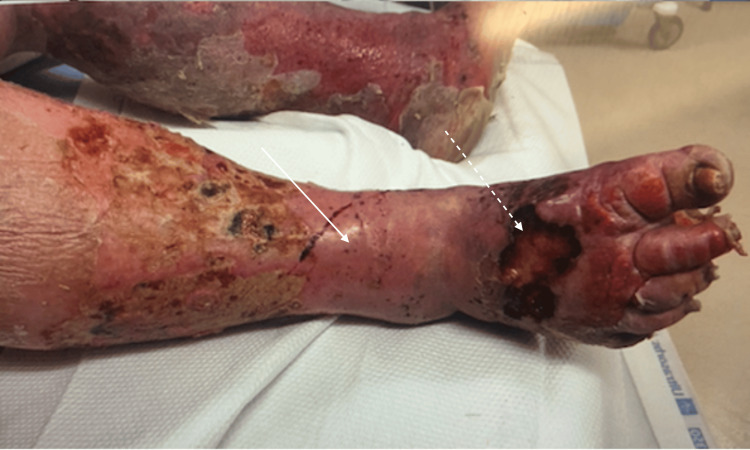
Taut, shiny skin (solid arrow) with hemorrhagic bullae and blackened eschar (dashed arrow)

**Figure 3:**
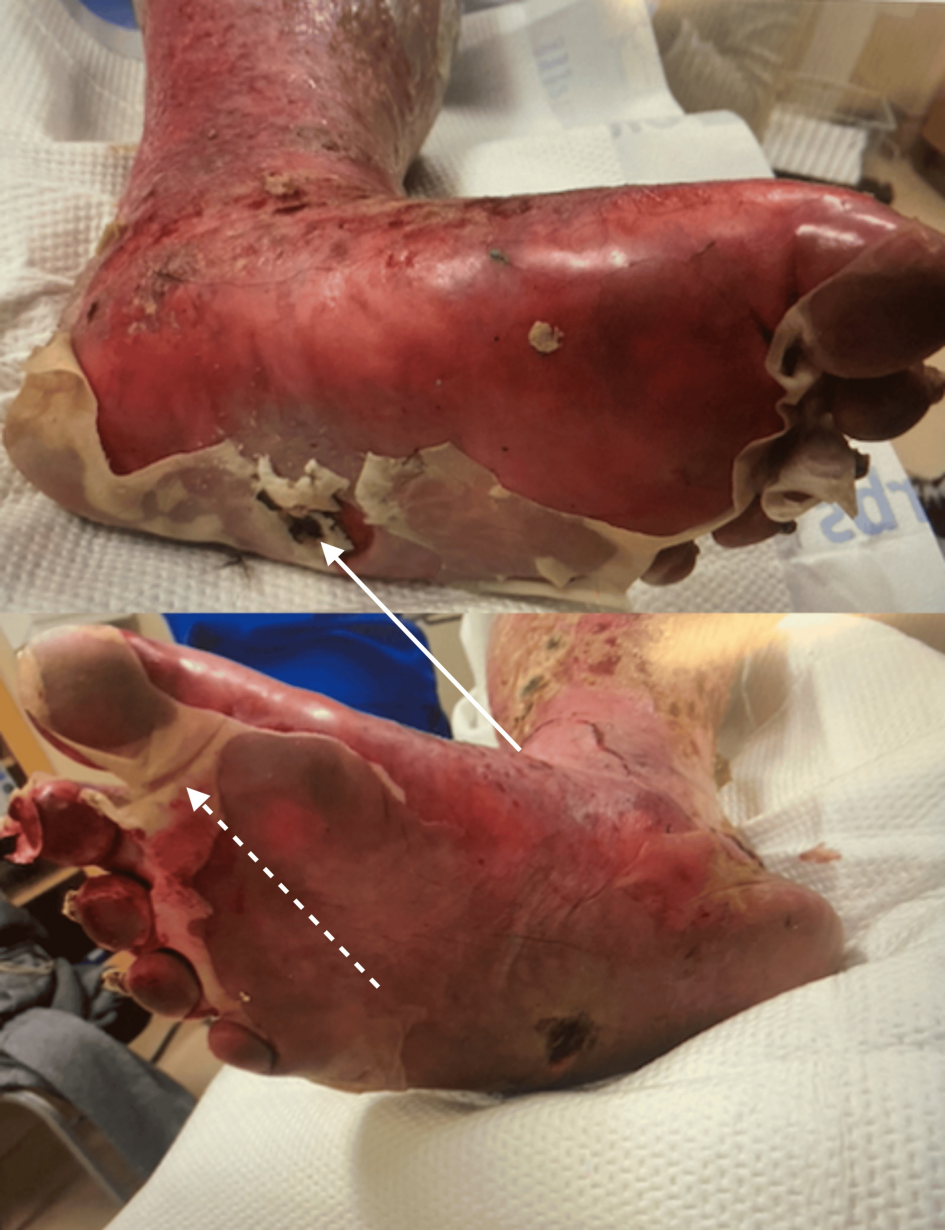
Necrotic debris (solid arrow) with epidermal sloughing and desquamation (dashed arrow)

On arrival, her temperature was 97 degrees Fahrenheit (36.1 degrees Celsius), heart rate 73 beats per minute, respiratory rate 17 breaths per minute, blood pressure 104/72 mmHg, and oxygen saturation 100% on pulse oximetry. She appeared chronically ill and had notably poor dentition. Extensive cutaneous and subcutaneous abnormalities involved the bilateral distal legs and feet. Diffuse erythema with violaceous and dusky discoloration mixed with gray-brown patches was circumferentially distributed around the ankles and feet. Marked edema and tense swelling distorted the normal anatomical contours of the feet and toes. Lipodermatosclerosis suggested a history of long-term venous insufficiency. Large areas of epidermal sloughing and desquamation exposed underlying erythematous and necrotic tissue. Multiple hemorrhagic bullae and blackened eschars were present, particularly over pressure points and the distal feet. Shiny and taut skin surrounded irregular ulcerations and necrotic debris. The nails appeared hypertrophic with darkened beds, suggesting chronic perfusion insufficiency. No observable crepitus was identified, and peripheral pulses were either diminished or palpation was blunted by significant edema. Diffuse tenderness was present, worsening as palpation progressed distally. Sensation and strength were intact bilaterally; however, pain limited the range of motion (Figures 1-3).

Laboratory samples were collected, including serum variables represented in the LRINEC score (Table 1).

**Table 1:** Laboratory analysis and LRINEC score LRINEC: Laboratory Risk Indicator for Necrotizing Fasciitis; GFR: glomerular filtration rate

Laboratory	Reported Values	Reference Range
White Blood Cell Count (WBC)	21.3 × 10^3^/µL	4.0-11.0 × 10^3^/µL
Hemoglobin (Hgb)	10.3 g/dL	12.0-16.0 g/dL
Sodium	138 mEq/L	135-145 mEq/L
Potassium	3.2 mEq/L	3.5-5.1 mEq/L
Blood Urea Nitrogen (BUN)	104 mg/dL	7-20 mg/dL
Creatinine	2.57 mg/dL	0.6-1.3 mg/dL
Estimated GFR	21 mL/min	>60 mL/min
Glucose	89 mg/dL	70-99 mg/dL (fasting)
C-reactive Protein (CRP)	10.2 mg/L	<3.0 mg/L
Procalcitonin	3.48 ng/mL	<0.10 ng/mL
Lactic Acid	1.9 mmol/L	0.5-2.2 mmol/L
LRINEC Score	5	<6 = low risk

Results showed elevated inflammatory markers, including a white blood cell count (WBC) of 21.3 × 10^3 µL (reference 4.0-11.0 × 10^3 µL) and a C-reactive protein (CRP) of 10.2 mg/L (reference <3.0 mg/L). Procalcitonin measured 3.48 ng/mL (reference <0.10 ng/mL), suggesting possible systemic inflammatory response and sepsis. Lactic acid was within normal range at 1.9 mmol/L (reference 0.5-2.2 mmol/L). Metabolic evaluation showed normal sodium at 138 mEq/L (reference 135-145 mEq/L), a hypokalemic potassium level of 3.2 mEq/L (reference 3.5-5.1 mEq/L), and a glucose of 89 mg/dL (reference 70-99 mg/dL fasting). Acute kidney injury was evident with a creatinine of 2.57 mg/dL (reference 0.6-1.3 mg/dL) and estimated glomerular filtration rate (GFR) of 21 mL/min (reference >60 mL/min). Mild anemia was noted with a hemoglobin (Hgb) of 10.3 g/dL (reference 12.0-16.0 g/dL).

Incorporating the available laboratory results, a LRINEC score of 5 was calculated, categorizing the patient as low risk and decreasing the likelihood of necrotizing fasciitis. Given the patient’s history and physical examination findings, a high index of suspicion was maintained for necrotizing fasciitis, and advanced imaging was attained.

**Figure 4:**
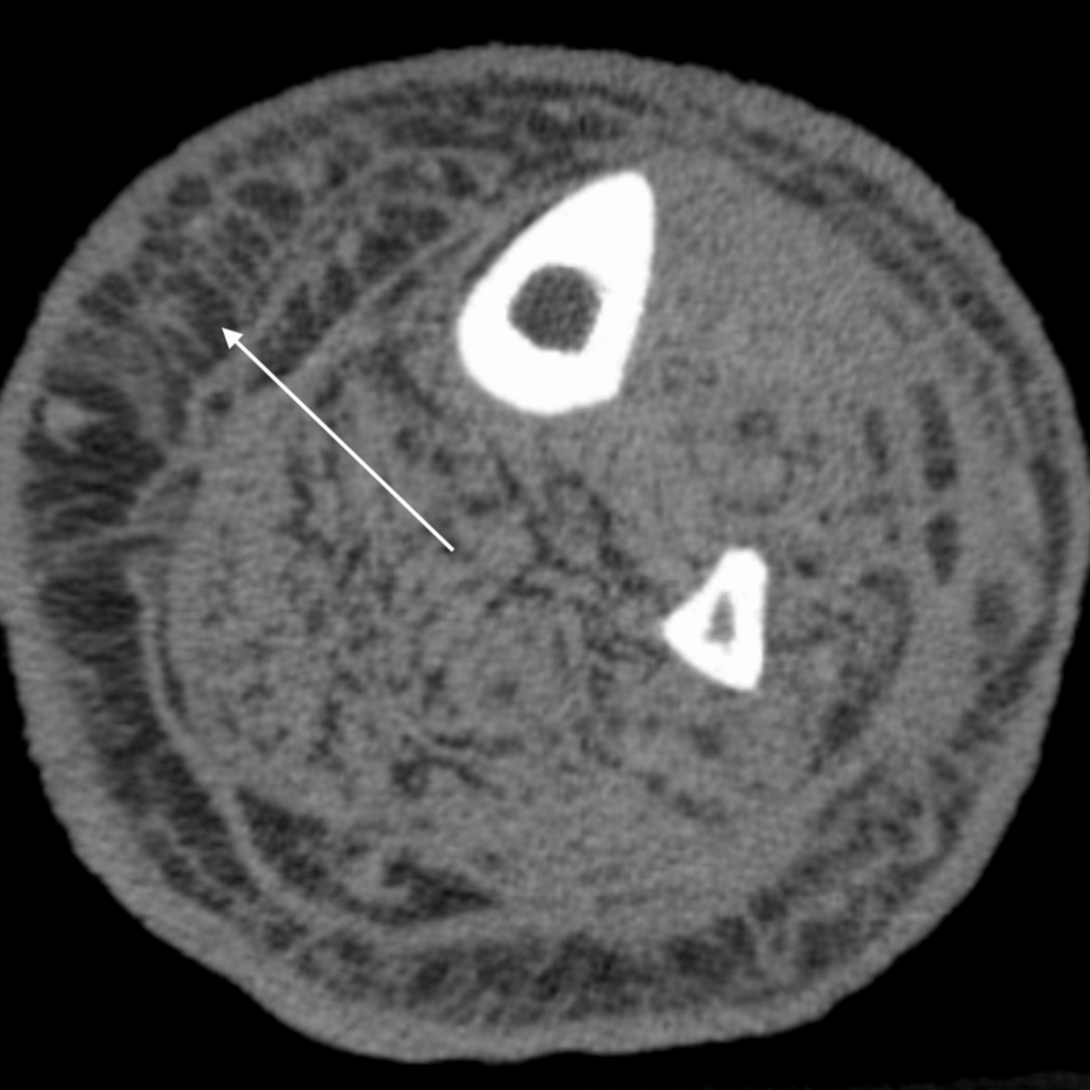
Axial non-contrast CT of the lower leg with extensive subcutaneous soft-tissue edema and stranding (arrow)

**Figure 5:**
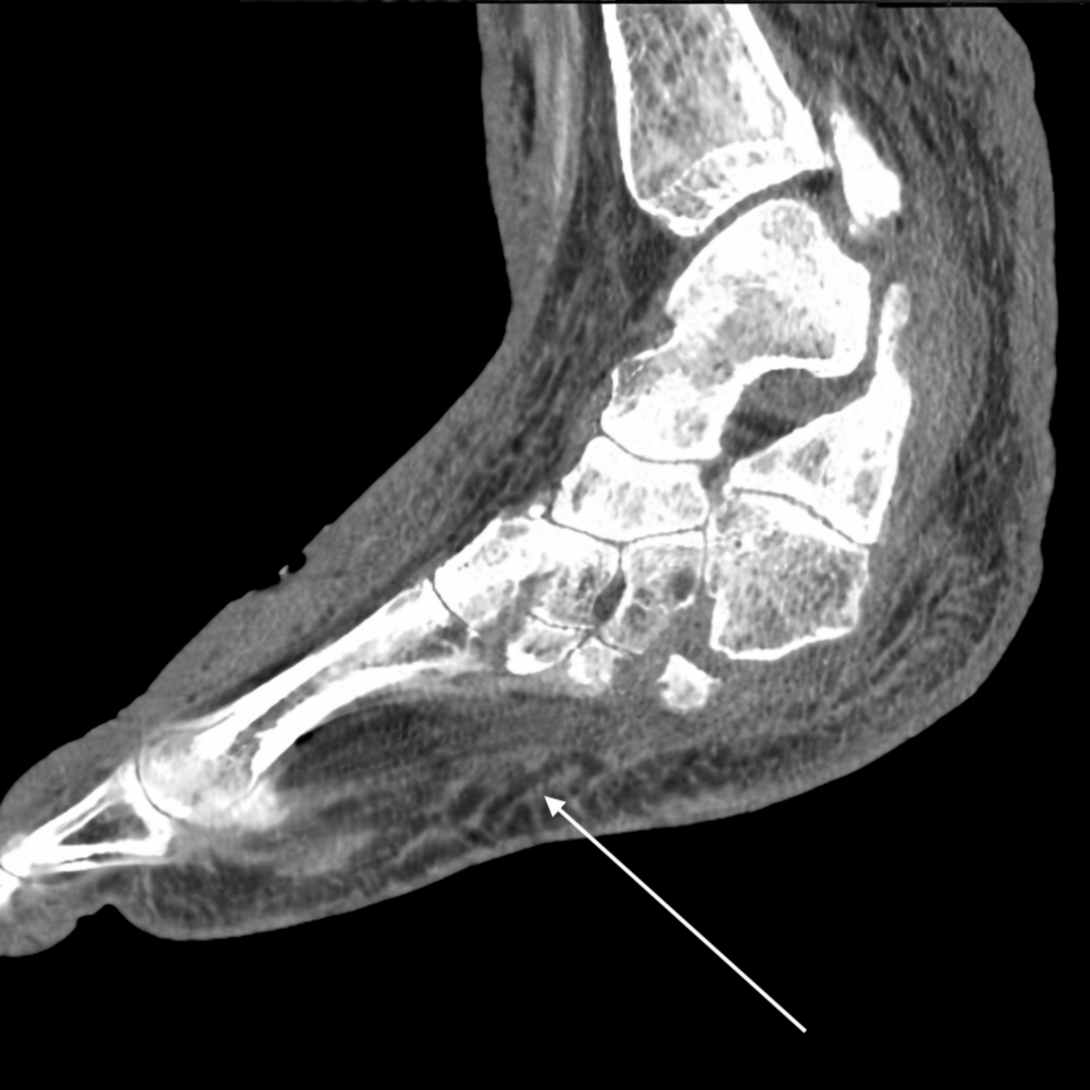
Sagittal non-contrast CT of the foot showing diffuse plantar soft-tissue edema (arrow)

**Figure 6:**
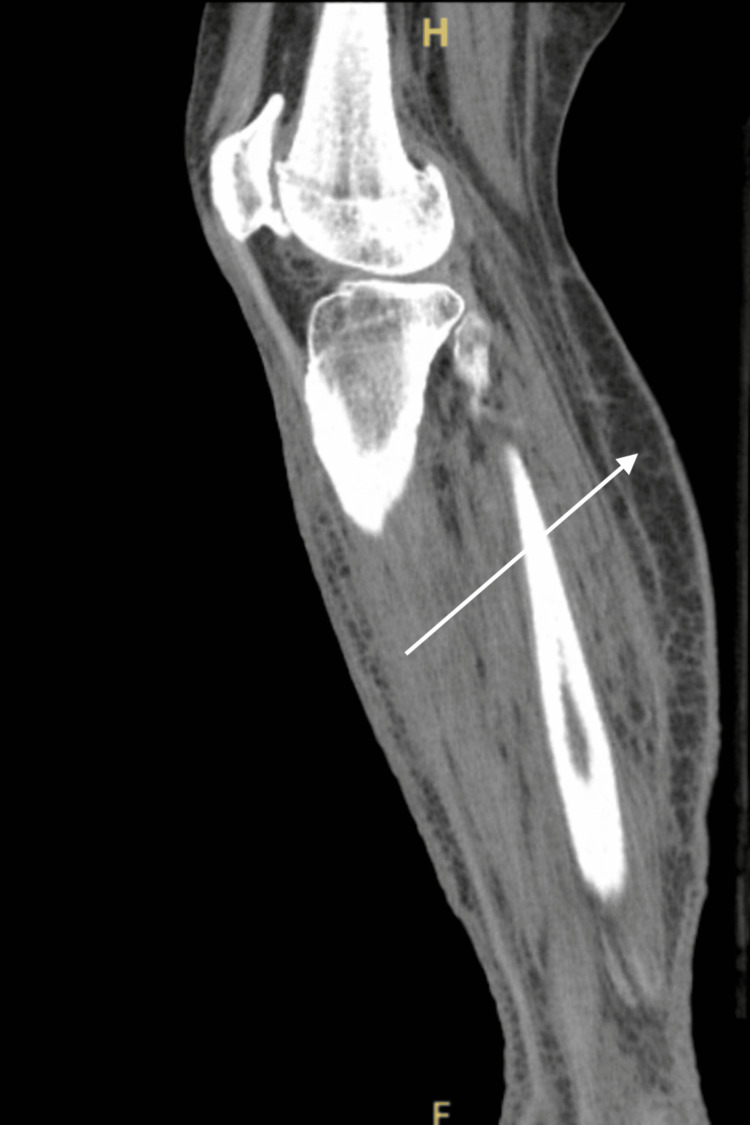
Sagittal non-contrast CT of the lower leg showing extensive soft-tissue edema (arrow)

Non-contrast computed tomography (CT) of the bilateral lower extremities demonstrated extensive soft-tissue thickening from the knees to the ankles and feet with prominent subcutaneous edema, stranding, inflammatory changes, and loss of the normal fascial planes. The radiologist did not confirm the presence of soft tissue gas or deep abscess formation (Figures 4-6).

The patient’s management prioritized early surgical consultation and intervention. A multidisciplinary team, including an infectious disease specialist and wound care, was assembled. Empiric broad-spectrum antibiotics were initiated with intravenous vancomycin and piperacillin-tazobactam to address the polymicrobial nature of necrotizing fasciitis. Adjunctive intravenous clindamycin was also ordered to curtail toxin formation. Treatment included liberal fluid resuscitation with normal saline and pain management with multimodal analgesia. General surgery promptly transported the patient to the operating room, where a bilateral below-the-knee amputation was performed. Purulent collections and extensive necrotic tissue were observed during the procedure. Intraoperative cultures were obtained, and methicillin-resistant *Staphylococcus aureus* (MRSA) was later isolated. Wound care interventions and nutritional support with strict glycemic control optimized postoperative healing. Physical therapy personnel provided rehabilitation and functional recovery. Social workers arranged housing resources and substance use counseling. Following hospitalization, she was discharged to a skilled nursing facility for ongoing wound management, comprehensive rehabilitation, and access to social support to facilitate overall recovery and address underlying social determinants of health.

## Discussion

Necrotizing fasciitis is a life-threatening, progressive soft tissue infection characterized by fascial necrosis and a toxic systemic inflammatory response resulting in high mortality, especially when the diagnosis and early directed therapy, including timely surgery, are delayed. Early history and physical examination findings may be subtle and nonspecific, making differentiation from cellulitis or other less aggressive soft tissue infections challenging. In response to necrotizing fasciitis’ propensity for diagnostic delay and the morbidity implications of protracted or indeliberate interventions, objective methods have been proposed to assist in prompt risk stratification. One such application, the LRINEC score, provided a promising, accessible, and attractive tool for expedient and accurate assessment of early soft tissue infection presentations.

The LRINEC score was initially proposed and investigated by Wong et al. in a 2004 retrospective observational study evaluating its utility as a novel system for early identification of necrotizing fasciitis in patients presenting with soft-tissue infections [1]. Several biochemical and hematologic variables were selected for their associations with the systemic inflammatory response and their ubiquitous availability in traditional laboratory evaluations (Table 2) [1].

**Table 2:** LRINEC score The Laboratory Risk Indicator for Necrotizing Fasciitis (LRINEC) score components were adapted from Wong et al. [1].

Laboratory Variables	Value Range	Points
C-reactive Protein (mg/L)	<150	0
C-reactive Protein (mg/L)	≥150	4
White Blood Cell Count (×10^3^/µL)	<15	0
White Blood Cell Count (×10^3^/µL)	15-25	1
White Blood Cell Count (×10^3^/µL)	>25	2
Hemoglobin (g/dL)	>13.5	0
Hemoglobin (g/dL)	11-13.5	1
Hemoglobin (g/dL)	<11	2
Sodium (mmol/L)	≥135	0
Sodium (mmol/L)	<135	2
Creatinine (mg/dL)	≤1.6	0
Creatinine (mg/dL)	>1.6	2
Glucose (mg/dL)	≤180	0
Glucose (mg/dL)	>180	1

The resulting score was used to risk-stratify patients into one of three risk groups: low, intermediate, or high (Table 3) [1].

**Table 3:** LRINEC risk stratification The Laboratory Risk Indicator for Necrotizing Fasciitis (LRINEC) score components were adapted from Wong et al. [1].

Total Score	Risk Category
0-5	Low
6-7	Intermediate
>8	High

The investigation found that an LRINEC score of 6 increased the likelihood of necrotizing fasciitis, and a score of 8 was highly predictive of necrotizing fasciitis [1]. Specifically, a score of 6 had a positive predictive value (PPV) of 92% and a negative predictive value (NPV) of 96% [1]. While sensitivity and specificity were not directly calculated by Wong et al., later authors reconstructed these numbers from the original data and frequently report a sensitivity of 0.92 and a specificity of 0.96 for an LRINEC score of 6 [6]. Wong et al. concluded that the LRINEC score, in conjunction with a thorough historical evaluation and physical examination, was a valuable tool for assessing clinically ambiguous soft-tissue infections, aiding early identification of necrotizing fasciitis, and prioritizing advanced imaging and early interventions for higher-scoring individuals [1]. The LRINEC score is a publicly available tool and does not require licensing or permission for use [1].

Given the score’s apparent utility in the early diagnosis of a notoriously difficult clinical dilemma, in which prompt recognition is a critical determinant of favorable outcomes, LRINEC was met with much enthusiasm and optimism. Subsequent validation studies with patient populations similar to the Wong et al. cohort seemed to confirm the accuracy of LRINEC and reinforce its promise as a rule-in and potential rule-out tool (Table 4) [2-5].

**Table 4:** Reported sensitivity and specificity estimates from LRINEC early literature * Sensitivity and specificity values for Wong et al. [1] were not directly reported in the original derivation study and were subsequently reconstructed by later validation studies. LRINEC: Laboratory Risk Indicator for Necrotizing Fasciitis

Study	Year	Study Design	Sensitivity (%)	Specificity (%)
Wong et al. [1]*	2004	Retrospective Cohort	92	96
Su et al. [2]	2008	Retrospective Cohort	89	90
Holland [3]	2009	Retrospective Cohort	80	67
Liao et al. [4]	2012	Retrospective Cohort	85	88
Colak et al. [5]	2014	Retrospective Cohort	84	87

These early supportive investigations, among others, were retrospective in design with relatively small sample sizes and observations of patients with advanced disease progression, factors that likely inflated diagnostic performance estimates of LRINEC’s inflammatory and metabolic laboratory markers and limited generalizability to early presentations [2-5].

More contemporary analysis has raised questions about LRINEC’s role and exposed potential limitations in its modern implementation. Later studies, including larger retrospective cohorts, prospective emergency department validations, and systematic reviews applied LRINEC to broader and more heterogeneous populations, including earlier and atypical presentations [6-10]. In contrast to early validation studies, several contemporary investigations report pooled diagnostic estimates derived from heterogeneous study populations, limiting direct comparability with earlier single-cohort validation studies [6-10]. These more recent investigations have consistently demonstrated markedly lower sensitivity than their earlier counterparts (Table 5) [6-10].

**Table 5:** Reported sensitivity and specificity estimates from contemporary LRINEC literature * Sensitivity and specificity values represent pooled estimates reported in systematic reviews and meta-analyses rather than single-study primary measurements. LRINEC: Laboratory Risk Indicator for Necrotizing Fasciitis

Study	Year	Study Design	Sensitivity (%)	Specificity (%)
Fernando et al. [6]*	2019	Meta-Analysis	68	85
Hsiao et al. [7]	2020	Prospective Cohort	43	83
Tarricone et al. [8]*	2022	Meta-Analysis	36	72
Hoesl et al. [9]	2022	Retrospective Cohort	48	79
Breidung et al. [10]	2023	Retrospective Cohort	59	82

**Figure 7:**
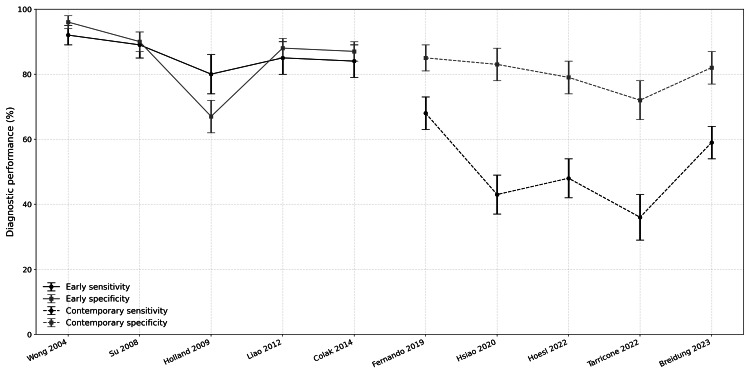
Diagnostic performance of the LRINEC score across early and contemporary studies Sensitivity and specificity values are derived from published estimates reported or reconstructed in the cited studies. Values for Wong et al. [1] were reconstructed in later validation analyses. Values for Fernando et al. [6] and Tarricone et al. [8] represent pooled estimates from meta-analyses. Pooled estimates are displayed for illustrative trend comparison and should not be interpreted as single-cohort diagnostic performance. LRINEC: Laboratory Risk Indicator for Necrotizing Fasciitis

Notably, a 2019 systematic review and meta-analysis by Fernando et al. documented pooled sensitivity estimates substantially lower than those in previous reports [6]. Hsiao et al. found a sensitivity of 0.43 for an LRINEC score of 6 in their emergency department population [7]. Tarricone et al. reported wide variability across studies and a pooled sensitivity estimate of 0.36 [8]. The trend of diminishing sensitivity is a departure from early validation studies, while specificity has remained more conservatively preserved than in initial investigations [6-10]. Some recent smaller prospective and observational investigations, such as Adhil et al. in 2023, have reported more favorable sensitivity values, but this is not representative of the overall progression [11]. The decline in sensitivity over successive validation studies can be explained by several methodological and epidemiological variables. Early studies predominantly evaluated hospitalized patients with advanced necrotic infection and systemic inflammation, in which laboratory abnormalities would be more pronounced and amplify LRINEC’s apparent diagnostic signal [1-5]. More recent studies relied on undifferentiated emergency department populations, earlier disease presentations, and a broader spectrum of soft-tissue infections [6-10]. Methodological rigor, including efforts to mitigate spectrum, incorporation, and timing biases, resulted in a model that demonstrated greater real-world diagnostic uncertainty and offered a more clinically representative framework for evaluation. Increased heterogeneity in patient populations, disease severity, and clinical presentation is reflected in the wider confidence intervals reported in contemporary studies, underscoring the limited precision and inconsistent performance of the LRINEC score across modern practice settings (Figure 7) [1-10].

Taken together, the evolving scholarship on LRINEC suggests diminished sensitivity and limited ability to safely exclude necrotizing fasciitis in early presentations of soft-tissue infections, but it may raise suspicion in the context of higher scores. Therefore, LRINEC may most appropriately fulfill a cautious adjunctive role in the assessment of possible necrotizing fasciitis, complementing rather than replacing clinical assessment, imaging, and surgical consultation.

Alternative assessment strategies have been proposed, including the Site, Immunosuppression, Age, Renal impairment, and Inflammatory markers (SIARI) calculator and a modified LRINEC score (m-LRINEC) that incorporates additional variables, such as serum lactate, historical findings, and comorbid conditions, and applies different laboratory cut-off values [12-16]. Individual laboratory markers, such as procalcitonin, have been investigated, as have advanced imaging modalities, particularly contrast-enhanced computed tomography and magnetic resonance imaging [17-19]. Point-of-care ultrasound has demonstrated utility as an adjunct to initial clinical assessment [20-22]. While many studies have shown promising improvements in accuracy over LRINEC, no alternative system has consistently demonstrated adequate sensitivity or been validated to safely rule out necrotizing fasciitis.

This report has several limitations. First, the literature review was focused rather than systematic, emphasizing frequently cited, high-impact studies to broadly illustrate the evolution of LRINEC’s performance over time. This method was chosen to contextualize the case report and may not capture the full breadth of published LRINEC investigations. Second, metrics such as sensitivity vary substantially across LRINEC research, likely secondary to study design, patient populations, disease severity at presentation, timing of laboratory testing, diagnostic reference standards, and divergent LRINEC cutoff values. In several instances, sensitivity and specificity values were derived from secondary analysis and pooled estimates rather than uniformly reported primary data points. While far from a meta-analytic systematic review, this report provides an accessible representation of the overall trend of LRINEC scholarship and highlights the score’s declining sensitivity and limitations as a rule-out tool for necrotizing fasciitis. The application of LRINEC is best relegated to an adjunctive role, supplementing but not replacing physician judgement and cautious clinical awareness.

## Conclusions

Necrotizing fasciitis remains a life-threatening condition in which delayed diagnosis can lead to significant morbidity and mortality. While laboratory-based scoring systems, including the LRINEC score, may contribute to early risk assessment, reliance on any single tool in isolation may be insufficient. This case and focused review underscore the importance of prioritizing clinical judgement, physical examination, prudent imaging, and early surgical consultation, while cautiously implementing LRINEC as an adjunct in the broader context of a comprehensive clinical assessment. Continued efforts to refine diagnostic strategies may enhance early recognition, but timely clinical decision-making remains paramount.
